# Comparing parasitological *vs* serological determination of *Schistosoma haematobium* infection prevalence in preschool and primary school-aged children: implications for control programmes

**DOI:** 10.1017/S0031182014000213

**Published:** 2014-03-28

**Authors:** WELCOME M. WAMI, NORMAN NAUSCH, KATHARINA BAUER, NICHOLAS MIDZI, REGGIS GWISAI, PETER SIMMONDS, TAKAFIRA MDULUZA, MARK WOOLHOUSE, FRANCISCA MUTAPI

**Affiliations:** 1 Institute of Immunology & Infection Research, University of Edinburgh, Ashworth Laboratories, King's Buildings, West Mains Rd, Edinburgh EH9 3JT, UK; 2 National Institute of Health Research, P.O. Box CY 573, Causeway, Harare, Zimbabwe; 3 Ministry of Health and Child Care, Murewa District Hospital, P.O. Box 60, Murewa, Zimbabwe; 4 Centre for Immunity, Infection & Evolution, University of Edinburgh, Ashworth Laboratories, King's Buildings, West Mains Rd, Edinburgh EH9 3JT, UK; 5 University of Zimbabwe, Biochemistry Department, P.O. Box MP167, Mount Pleasant, Harare, Zimbabwe

**Keywords:** parasitology, serology, prevalence, schistosomiasis, diagnosis, neglected tropical diseases

## Abstract

To combat schistosomiasis, the World Health Organization (WHO) recommends that infection levels are determined prior to designing and implementing control programmes, as the treatment regimens depend on the population infection prevalence. However, the sensitivity of the parasitological infection diagnostic method is less reliable when infection levels are low. The aim of this study was to compare levels of *Schistosoma haematobium* infection obtained by the parasitological method *vs* serological technique. Infection levels in preschool and primary school-aged children and their implications for control programmes were also investigated. Infection prevalence based on serology was significantly higher compared with that based on parasitology for both age groups. The difference between infection levels obtained using the two methods increased with age. Consequentially, in line with the WHO guidelines, the serological method suggested a more frequent treatment regimen for this population compared with that implied by the parasitological method. These findings highlighted the presence of infection in children aged ⩽5 years, further reiterating the need for their inclusion in control programmes. Furthermore, this study demonstrated the importance of using sensitive diagnostic methods as this has implications on the required intervention controls for the population.

## INTRODUCTION

Urogenital schistosomiasis is a waterborne disease caused by infection with *Schistosoma haematobium* and is a major public health problem among poor communities in sub-Saharan Africa (Gryseels *et al.* 2006; Kabatereine *et al.* 2007; WHO, 2012). Eggs laid by adult female *S. haematobium* worms are excreted through urine, inflicting damage to the genitourinary tract. Children living in endemic areas tend to carry the highest disease burden (Hotez *et al.* 2006; Stothard *et al.* 2011*a*) and symptoms of urogenital schistosomiasis amongst these children are commonly characterized by the presence of blood in urine (haematuria) and painful urination (van der Werf *et al.* 2003; Sady *et al.* 2013). Chronic infection results in severe pathologies such as kidney failure and urinary tract and bladder wall fibrosis. Other symptoms include malnutrition, stunted growth and impaired memory and cognition (Pasvol and Hoffman, 2001; Sousa-Figueiredo *et al.* 2008; WHO, 2010; Muller *et al.* 2011).

The infection and its associated morbidity can be controlled with chemotherapy using praziquantel (Doenhoff *et al.* 2008; Mutapi *et al.* 2011), administered at a standard oral dosage of 40 mg kg^−1^ body weight (WHO, 2002). Praziquantel is safe and efficacious in children aged 5 years or under (Mutapi *et al.* 2011; Stothard *et al.* 2011*b*; Coulibaly *et al.* 2012), but so far treatment of children belonging to this age group has not yet been fully integrated into the control programmes (Ekpo *et al.* 2012). Preschool-aged (⩽ 5 years) children have been neglected both in terms of research and in control programmes for the previously held view that they carry insignificant infection levels (Stothard and Gabrielli, 2007; Mutapi *et al.* 2011; WHO, 2011). This was further exacerbated by poor diagnosis of infection in the field (Vennervald *et al.* 2000; Stothard *et al.* 2011*a*). The exclusion of preschool-aged children from current control programmes increases their risk of developing future morbidity (Stothard and Gabrielli, 2007; Sousa-Figueiredo *et al.* 2008) and also indicates that disease burden in this age group is still not well defined (Garba *et al.* 2010). Consequently, this may have negative impacts on the overall effectiveness of control programmes.

In line with the guidelines outlined by the World Health Organization (WHO, 2002), infection prevalence must be determined prior to the implementation of a control programme (Dawson *et al.* 2013; WHO, 2013). To ensure that infection transmission levels are reduced and the associated morbidity is alleviated, repeated mass drug administration (MDA) at regular intervals depending on the population prevalence has been recommended by the WHO (Hotez *et al.* 2006; Kabatereine *et al.* 2007; WHO, 2013). Thus, it is important that sensitive diagnostic tools are applied to determine infection levels in the population.

Egg count in urine (parasitology) is the widely accepted approach for quantifying *S. haematobium* infection levels in a population (WHO, 1998; Pasvol and Hoffman, 2001; Kinkel *et al.* 2012). However, the parasitological method is less sensitive in light infections (Doenhoff *et al.* 2004; Bergquist *et al.* 2009). Furthermore, parasitology does not diagnose pre-patent or single-sex infections where there is no egg production (Mutapi, 2011). Several additional methods aimed at improving the diagnosis of schistosomiasis have been evaluated (Stothard *et al.* 2013), although the focus has been mainly on *Schistosoma mansoni* (Sorgho *et al.* 2005; de Noya *et al.* 2007; Stothard *et al.* 2011*a*). Examples of additional diagnostic methods include antibody detection (Sorgho *et al.* 2005; de Noya *et al.* 2007; Smith *et al.* 2012), dipstick detection of haematuria (Adesola *et al.* 2012; King and Bertsch, 2013) and use of reported questionnaires about presence of haematuria (Lengeler *et al.* 2002; Clements *et al.* 2008). There is currently a paucity of studies comparing different methods of detecting infection in preschool-aged children. The elegant dipstick meta-analysis study recently published by King and Bertsch (2013) highlights the need for more investigations on different methods for detecting infection in preschool-aged children.

The first aim of our study was to compare levels of *S. haematobium* infection determined by the parasitological method with infection detected via the serological technique and their implications for the WHO recommended treatment regimens for this study population. Dipstick microhaematuria was also used as an additional tool to the parasitological method on a subset of this study population to detect *S. haematobium* infection. The second aim of this study was to determine infection levels in preschool-aged children in comparison to primary school-aged children to elucidate the implications of these levels of infection for childhood health and their inclusion in the current control programmes.

## MATERIALS AND METHODS

### Ethical approval and consent

The study received ethical and institutional approval from the University of Zimbabwe and the Research Council of Zimbabwe. Permission to conduct the work in this province was obtained from the Provincial Medical Director, the District Educational Officer and Heads of schools in the study area. Project aims and procedures were fully explained to the community, primary school-aged children, teachers and parents/guardians in the local language, Shona. Written informed consent/assent was obtained from parents/guardians prior to enrolment of children into the study. The children were recruited into the study on a voluntary basis and were free to withdraw at any time with no further obligation. Children in this study were offered treatment with the standard dose of praziquantel administered by the local physician.

### Study area and population

The study was conducted in two rural villages in Murewa district, in the north-east of Zimbabwe (31°90′E; 17°63′S). The area is a high *S. haematobium* transmission area according to the WHO classification of having a prevalence of infection >50% (WHO, 2002). Prevalence of *S. mansoni* and soil transmitted helminths (STH) is low in this area (Ndhlovu *et al.* 1996; Nausch *et al.* 2012). The children were recruited from crèches, early child development centres, preschools (typically for 3–5 years old) and local primary schools (for 6–10 years old). Parents/guardians with children not attending any of the education programmes (e.g. children <3 years old) in the area were invited to report to the school centre for enrolment into the project.

### Study design

The inclusion/exclusion criteria for this study were as follows: children should have (1) been lifelong residents of the study area; (2) had no prior history of anthelmintic treatment (the above two criteria were assessed by means of questionnaires administered to parents/guardians for all children); (3) had provided at least 3 urine samples for *S. haematobium* detection and 2 stool samples for STH and *S. mansoni* parasitological examination; (4) been negative for *S. mansoni* infection (21 children were excluded from the study based on this criterion); and (5) been negative for STH infections (no children were excluded based on this criteria as no STH were detected in any of the participants). A total of 438 children (54·6% females and 48·9% males) with complete parasitological and serological data were available for investigation in this study (Table A1). Of the surveyed children, 224 (51·1%) resided in village 1 and 214 (48·9%) were residents of village 2.

### Parasitology

Urine samples collected on 3 consecutive days were examined microscopically for *S. haematobium* infection using the standard filtration method (Mott *et al.* 1982). *Schistosoma mansoni* infection was diagnosed from stool samples collected on 2 consecutive days using the Kato-Katz method (Katz *et al.* 1972). Children were designated infected with *S. haematobium* if at least one egg was detected in any of their urine samples and similarly for *S. mansoni* with a single egg detected in stool. The *S. haematobium* infection intensity was calculated using the arithmetic mean egg count per 10 mL of the collected urine samples. For very young children where it was difficult to obtain samples on the spot, the samples were collected overnight by parents/guardians using urine collection bags (Hollister 7511 U-Bag Urine Specimen Collector, Hollister Inc., Chicago, IL, USA) and stool samples were collected using disposal dippers.

### Serology

Serum was obtained from up to 5 mL of venous blood collected from each child, frozen at −20 °C in the field and transferred to a −80 °C freezer in the laboratory, prior to shipment to the University of Edinburgh, UK and kept under storage at −80 °C. Samples were thawed for the first time for use in this study. The sera were tested for IgM (Dako, UK) antibody responses directed against schistosome egg antigens using enzyme linked immunosorbent assays (ELISA). The ELISA were conducted in duplicate per plate as previously described (Mutapi *et al*. 1997; Imai *et al.* 2011). The results were expressed as the mean optical density (OD) value of the duplicate assay. IgM antibodies are produced early in an infection (Warrington *et al.* 2011) and previous studies have reported a positive association between anti-egg IgM antibody responses and schistosomiasis infection levels (Mutapi *et al.* 2003; Stothard *et al.* 2011*a*; Dawson *et al.* 2013). Thus, for this study we used anti-schistosome egg IgM antibody response as an additional diagnostic indicator for *S. haematobium* infection.

A total of 17 serum samples comprised of four serum samples from age-matched schistosome naïve European and 13 healthy Zimbabwean donors (schistosome infection-free and with no anomalies reported after clinical examination by the paediatrician) were used as controls to determine cut-off ELISA values for ‘infection’ status. The European samples were drawn from the Edinburgh anonymized clinical sample archive. The cut-offs were calculated using the formula: mean (OD) +2*standard deviations of the mean (s.d.). Children were classified as infected if their levels of parasite-specific antibody levels were greater than the cut-off value, and infection negative if equal or below the cut-off value.

### Dipstick microhaematuria

Out of the 438 children, 190 (51 preschool-aged and 139 primary school-aged) children in this study population had their urine samples examined for microhaematuria detectable by Uristix^®^ reagent strips (Plasmatec, UK) as an indicator for *S. haematobium* infection in addition to the parasitological and serological diagnostic methods (Table A2). No marked variability was noted in dipstick tests of the urine samples for each child collected on consecutive days, thus, only the dipstick test results for urine samples collected on the first day of the survey were used in this study. The levels of dipstick microhaematuria were first graded semi-quantitatively as: negative (−), single positive (+; ≈10 erythrocytes *μ*L^−1^), double positive (++; ≈50 erythrocytes *μ*L^−1^) and strong positive (+++; ≈250 erythrocytes *μ*L^−1^) following the manufacturer's guidelines. In this study, a positive test for microhaematuria was indicative of the presence of *S. haematobium* infection, meaning that children with scores of a single + and above were scored as positive for microhaematuria. A random sample of 123 urine samples were tested using the Multistix^®^ 10SG (Bayer, UK) in addition to the Uristix^®^ test to assess for differences in the quality of dipsticks by manufacturer. A strong agreement between the dipstick results from the two manufacturers using the McNemar's test (*P*<0·001) was observed, hence no evidence of the influence of the dipstick source on test results was noted for this study population.

### Statistical analyses

Statistical analyses were performed using SAS^®^ 9.3 (SAS Institute Inc., Cary, NC, USA) and R 3.0.1 (R Development Core Team, Vienna, Austria). Infection intensity was log-transformed (log_10_[egg count +1]) to meet the underlying assumptions of parametric statistical tests. Pearson's partial correlation coefficient (*r*) was used to measure the strength of the association between infection intensity and antibody levels, controlling for the effect of age. To investigate whether the mean antibody levels or mean infection intensity differed significantly between preschool and primary school-aged children, independent *t* tests were used. The effect of sex, age group and village on the mean infection intensity and on the antibody levels was investigated using general linear regression models. To determine whether infection prevalence differed between the two age groups and that prevalence derived from parasitological data differed from that based on serological data, Chi-square (*χ*^2^) tests were used.

### Age-dependent prevalence model

Infection prevalence based on the binary response variables derived from parasitology and serology as a function of age, was estimated parametrically using the method of generalized linear regression modelling. Letting *n* be the sample size under investigation, *a*_*i*_ the age of the *i*th child (*i* = 1, … *n*) and *q*(*a*) the proportion of infection-negative children at age *a* in the study population. The prevalence, which is the probability of being infected at age *a*, is given by: π(*a*)  = 1−*q*(*a*) and estimated using the binary response variable *Y*_*i*_ as follows: π(*a*) = *P*(*Y*_*i*_ = 1|*a*_*i*_). The generalized linear model with a complementary log-log link was applied to take into account the binary nature of the response (Mathei *et al.* 2006) and expressed parametrically as follows:
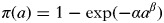
where *α* is the intercept and *β* is the slope, i.e. the coefficient representing the effect of age on the probability of being infection positive.

*P* values less than 0·05 were considered statistically significant in this study.

## RESULTS

### Infection intensity and antibody levels

The observed overall mean *S. haematobium* infection intensity based on egg counts was 17·40 eggs/10 mL urine (s.d. = 71·20) and the overall mean antibody levels was 0·62 OD (s.d. = 0·34). The mean infection intensity and antibody levels were significantly higher for the primary-school aged children compared with that for preschool-aged children as shown in Table 1. A large variability in egg counts was observed as indicated by the large s.d. of the mean for both age groups in Table 1. Based on parasitology, 7·1% of all children participating in this study carried heavy infections (⩾50 eggs/10 mL urine), 30·4% had light infections (1–49 eggs/10 mL urine) and 62·6% had no infection burden (0 eggs/10 mL urine) according to the WHO classes of infection intensity (WHO, 2002). Among the preschool-aged children, 3·1% had heavy infections, 15·5% had light infections and 81·4% had no infection and in primary school-aged children, 8·2% had heavy infections, 34·6% had light infections and 57·2% had no infection.

**Table 1.** Summary results for infection intensity using egg count per 10 mL urine, IgM antibody response in optical densities (OD) directed against schistosome egg antigens with standard deviation of the mean (s.d.) and *t*-test (on transformed data for infection intensity) for mean difference between the two age groups

Variable	Age group	*n*	Mean (s.d.)	Median	Minimum	Maximum	*t*	*P* value
Egg count	1–5 years	97	9·03 (47·53)	0·00	0·00	380·33	−4·49	<0·001
	6–10 years	341	19·78 (76·50)	0·00	0·00	1013·00		
Antibody level	1–5 years	97	0·46 (0·31)	0·38	0·01	1·27	−5·72	<0·001
	6–10 years	341	0·67 (0·33)	0·66	0·07	2·39		

Both infection intensity (*r* = 0·18; *P*<0·001) and antibody levels (*r* = 0·31; *P*<0·001) increased significantly with age. In addition, a positive correlation between infection intensity and antibody levels was found (*r* = 0·23; *P*<0·001). Infection intensity or antibody levels were not associated with sex and village of origin allowing for the effect of age (Table 2).

**Table 2.** *F* and *P* values from general linear regression models to test for the difference in mean infection intensity (transformed using log10[egg count +1]) and IgM antibody response directed against schistosome egg antigens by sex and village, adjusting for the effect of age

	Infection intensity	Antibody level
Variable	*F* (*P* value)	*F* (*P* value)
Age (years)	13·92 (<0·001)	47·47 (<0·001)
Sex (F *vs* M)	2·11 (0·147)	1·51 (0·220)
Village (1 *vs* 2)	0·49 (0·483)	0·48 (0·491)

### Infection prevalence: parasitology vs serology

**Fig. 1.**
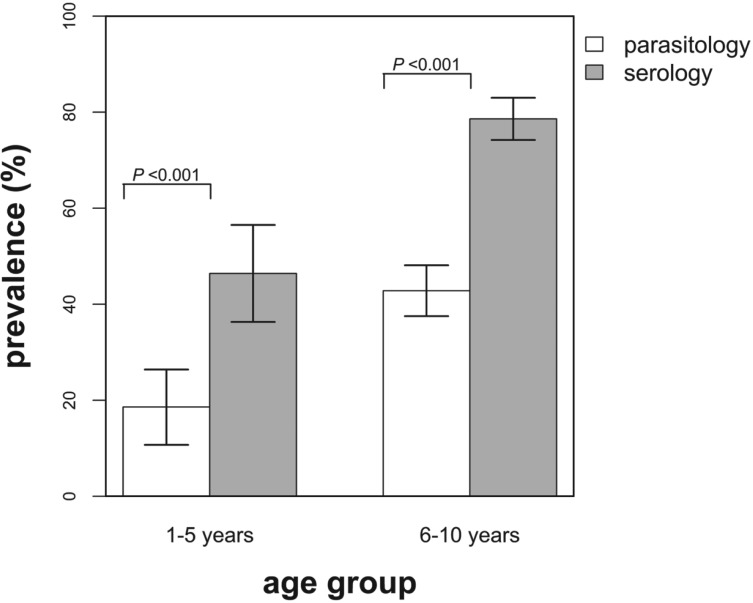
Infection prevalence derived using parasitological and serological diagnostic methods by age group. The indicated bars are the 95% confidence intervals of the observed prevalence and the *P* values test for the differences in prevalence between the diagnostic methods for each age group. White bars = prevalence based on parasitology and grey bars = prevalence based on serology.

The overall observed infection prevalence based on the two diagnostic techniques was as follows: parasitology, 37·4% (95% CI: 33·0–42·0%) and serology, 71·5% (95% CI: 67·2–75·7%) and these differed significantly (*χ*^2^ = 102·12; *P*<0·001). In addition, the infection prevalence based on serology was found to be significantly higher than the prevalence derived from parasitology for both age groups (Fig. 1). No significant difference in infection prevalence between male and female children was observed (parasitology, *χ*^2^ = 0·79; *P* = 0·374, and serology, *χ*^2^ = 0·15; *P* = 0·703).

**Fig. 2.**
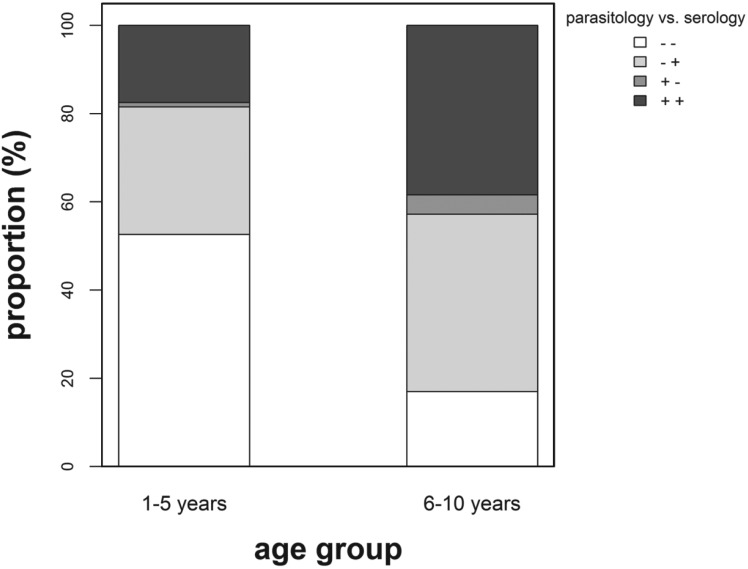
Percentage proportion positive (+) *vs* negative (−) children diagnosed using parasitological and serological methods by age group. White stack: (− −) = negative for both diagnostic methods (1–5 years, n = 51; 6–10 years, *n* = 58), light grey stack (− +) = negative for parasitology but positive for serology (1–5 years, *n* = 28; 6–10 years, *n* = 137), grey stack (+ −) = positive for parasitology but negative for serology (1–5 years, *n* = 1; 6–10 years, *n* = 15) and dark grey stack (+ +) = positive for both diagnostic methods (1–5 years, *n* = 17; 6–10 years, *n* = 131).

The proportion of children classified as infection negative using the parasitological technique in preschool-aged children was significantly lower (*χ*^2^ = 4·11; *P* = 0·043) compared with that in primary school-aged children (Fig. 2). For this study, only 16 (3·7%) children (10 female and 6 male, age ⩾5 years) were found egg positive but classified as infection negative using the serological diagnostic method. In addition, for 9 of these children, eggs were detected only in one urine sample, with a mean count of 4 eggs/10 mL urine or less.

### Age-dependent prevalence profiles: parasitology vs serology

**Fig. 3.**
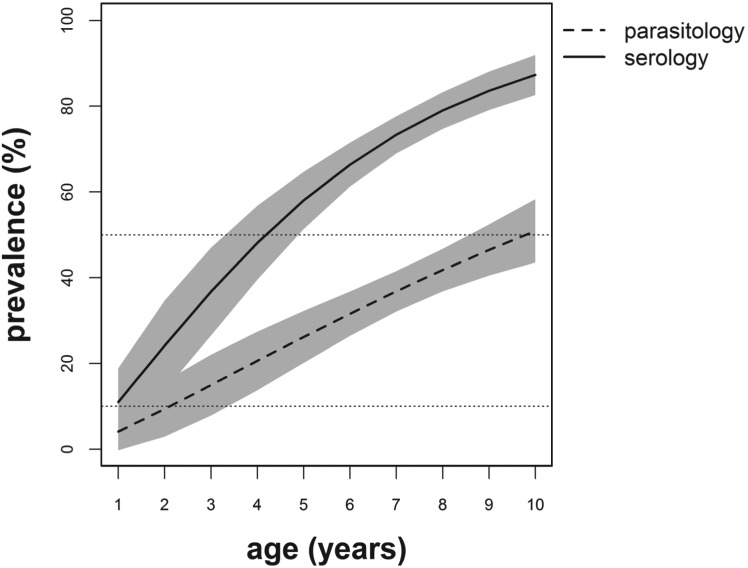
Predicted age-related infection prevalence profiles derived from parasitological (dashed line) and serological (solid line) diagnostic methods. The grey shadings around the prevalence curves indicate the 95% confidence intervals. The horizontal dashed lines indicate the moderate (10%) and high (50%) infection-risk cut-offs for control regimens as defined by the World Health Organization (WHO, 2002).

The results for estimated regression coefficients and s.e. used to determine the age-dependent infection prevalence based on parasitological *vs* serological data were as follows: intercept, *α*: 0·04 (s.e. = 0·02) *vs* 0·12 (s.e. = 0·05) and slope, *β*: 1·23 (s.e. = 0·27) *vs* 1·25 (s.e. = 0·19). Infection prevalence increased with age in a similar pattern for both diagnostic methods, however the rate of increase for serology was higher compared with that of parasitology (Fig. 3). In addition, the infection prevalence derived using the serological technique was higher compared with the prevalence based on parasitological diagnostic method and this discrepancy increased with age (Fig. 3). The infection levels for primary school-aged children based on serology belonged to the high-risk WHO category (prevalence ⩾50%) compared with the moderate-risk category implied by the parasitological diagnostic method.

### Dipstick microhaematuria diagnostic method

**Fig. 4.**
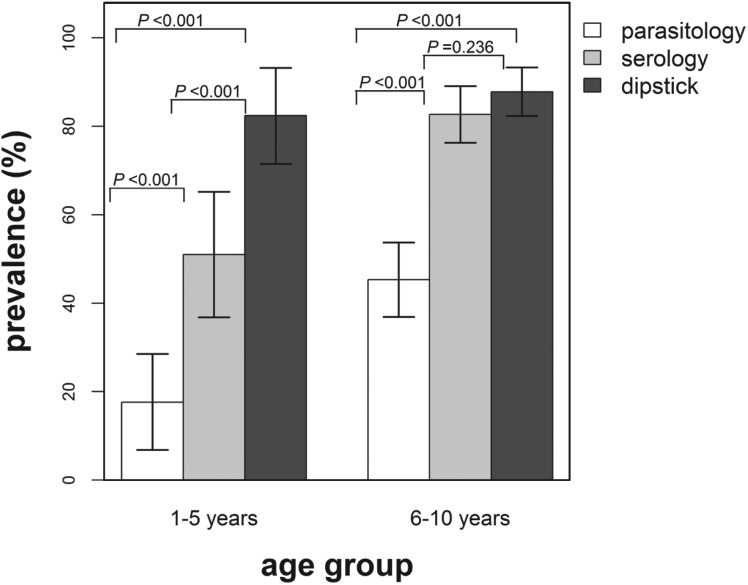
Infection prevalence derived using parasitological, serological and dipstick microhaematuria diagnostic methods by age group for a subset of the study population (*n* = 190). The indicated bars are the 95% confidence intervals of the observed prevalence and the *P*-values test for the differences in prevalence between the diagnostic methods for each age group. White bars = prevalence based on parasitology, grey bars = prevalence based on serology and dark grey bars = prevalence based on dipstick microhaematuria.

Infection prevalence derived using the dipstick microhaematuria test was compared with the infection prevalence determined using the parasitological diagnostic method on 190 children (Fig. 4). The overall infection prevalence derived from the dipstick microhaematuria in this subset of the study population was 86·3% (95% CI: 81·9–91·7%) compared with 37·9% (95% CI: 30·9–44·9%) based on parasitology and 74·2% (95% CI: 67·9–80·5%) derived from the serological diagnostic method. Furthermore, infection prevalence based on dipstick microhaematuria was significantly higher compared with prevalence based on parasitology for both age groups. It was further noted that none of the egg-positive children were diagnosed as infection negative using dipstick microhaematuria and 4 (2·9%) primary school-aged children were found egg positive but with no microhaematuria detected in urine.

## DISCUSSION

Following successful advocacy by the World Health Assembly (WHA, 2001), repeated MDA has become the key control strategy to combat schistosomiasis (WHO, 2002, 2013), with frequency of treatment dependent on the pre-determined infection prevalence (WHO, 2002). However, taking into consideration the reduced sensitivity of the parasitological diagnostic technique in children carrying light infections (Engels *et al.* 1997; Coulibaly *et al.* 2013), it is imperative that additional sensitive diagnostic tools are incorporated to improve the determination of infection levels. In this study we compared levels of *S. haematobium* infection obtained by the parasitological (egg count) method to the serological technique. In addition, these infection levels were compared between preschool and primary school-aged children to elucidate the need for inclusion of the neglected preschool age group into control programmes. The implications of infection levels determined in this study for the WHO recommended MDA regimens were also investigated.

In agreement with other studies using different diagnostic tools (Kahama *et al.* 1998; Kanamura *et al.* 2002; Lengeler *et al.* 2002; van Dam *et al.* 2004), infection levels (infection intensity and prevalence) increased significantly with age in this study. Unsurprisingly, infection intensity was positively correlated with anti-egg IgM antibody levels, since children accumulate infection with the associated increase in exposure to schistosome antigens (Stothard *et al.* 2011*b*). More importantly, the results of this study revealed significant infection prevalence in preschool-aged children, further concurring with findings from recent studies on the infection burden in this age group (Garba *et al.* 2010; Sousa-Figueiredo *et al.* 2010; Mutapi *et al.* 2011; Stothard *et al.* 2011*a*). These findings implicate a risk of preschool-aged children developing severe pathology due to chronic infection if left untreated (Stothard *et al.* 2011*b*; Ekpo *et al.* 2012). Hence the inclusion of these children in control programmes should be considered fundamental for improved and balanced childhood health (Garba *et al.* 2010).

This study revealed, in contrast with serology, that the parasitological technique approach underestimated infection prevalence in both age groups. These findings are indicative of reduced sensitivity of the parasitological technique since the majority of children in our study population carried light infection. In addition, following the WHO guidelines (WHO, 2002), infection prevalence derived from the serological method suggested a more frequent treatment intervention for this study population compared with that implicated by the parasitological technique. These findings further demonstrate that the use of different diagnostic techniques can be of importance in decision-making about suitable control strategies to implement. The WHO system is based upon parasitology, and was developed before the contribution of light infections (not detected via egg counts) to pathology was fully realized. The combination of additional diagnostics which can detect low infection levels and better definition of morbidity arising from low infections in *S. haematobium* infections (as recently summarized by King and Bertsch, 2013) support the current efforts for including preschool-aged children in schistosomiasis control programmes (Stothard *et al.* 2013).

The small proportion of schistosome egg-positive children in this study who were classified as infection negative using the serological technique can theoretically be attributable to two reasons: (1) contamination of the urine samples (Mutapi, 2011), which can occur as a result of instruments not being thoroughly cleaned or urine contamination with stool, especially for young female children; and (2) individual variability in mounting an immune response against the parasite antigens (Stothard *et al.* 2011*a*).

Similar patterns of age-dependent infection prevalence profiles were observed for both diagnostic methods, indicative of early exposure to infection and the accumulation of infection as children grow older (Garba *et al.* 2010; Stothard *et al.* 2011*a*). Overall, the estimated age-dependent prevalence based on serology was higher compared with that derived from parasitology, and this discrepancy between infection levels obtained from the two diagnostic methods also increased with age. Consequentially, the observed age-prevalence patterns indicated that the required intervention strategies varied with age.

The use of dipstick microhaematuria in this study detected higher prevalence of infection equally for both preschool and primary school-aged children in comparison to the parasitological method. These findings highlighted the usefulness of dipstick microhaematuria as an additional diagnostic tool in children carrying light infections, in agreement with findings from other recent studies (King and Bertsch, 2013). Haematuria due to glomerular causes has been reported in children (Meyers, 2004), thus caution should be exercised when interpreting the high prevalence of microhaematuria in preschool-aged children. Further studies are needed to elucidate levels of haematuria attributable to schistosome infection in this age group. French *et al.* (2007) recommended comparison of dipsticks sourced from different manufacturers to assess the effect of quality on the test results. In this study we used dipsticks sourced from two different companies (Uristix^®^ from Plasmatec and Multistix^®^ from Bayer) and they gave comparable results, supporting the robustness of our findings.

## CONCLUSION

In conclusion, this study showed significant *S. haematobium* infection levels among untreated preschool and primary school-aged children who were life-long residents of an endemic area. Infection intensity and prevalence increased rapidly from early childhood, highlighting the need for treatment of the preschool-aged children. This study further highlighted the essential need for incorporating preschool-aged children into control programmes for the health benefits of treatment currently being offered to their older counterparts and thus prevent creating a childhood health inequity (Mutapi *et al.* 2011; Stothard *et al.* 2011*b*, 2013). Infection prevalence based on serology suggested a more frequent MDA regimen to that implied by the parasitological technique. We reiterate the importance of using sensitive diagnostic methods to improve accuracy in estimating true infection prevalence as this has implications on the required MDA regimen for the population. In our study, serology was highlighted as a valuable sensitive diagnostic tool that could be applied in conjunction with the parasitological technique. The findings of this study revealed that dipstick microhaematuria was equally sensitive in diagnosing infection in both preschool and primary-school aged children. Further evaluation of detection of microhaematuria using dipsticks as an additional diagnostic tool for *S. haematobium* infection in preschool-aged children is recommended.

## APPENDIX

**Table A1.** Description of the different sample sizes of the study population (total, *n* = 438) for parasitology and serology data by age group

Age group	Parasitology	Serology		Total
		Negative	Positive	
1–5 years	Negative	51	28	79
	Positive	1	17	18
6–10 years	Negative	58	137	195
	Positive	15	131	146
	Total	125	313	438

**Table A2.** Description of the different sample sizes of the subset (total, *n* = 190) of the study population for parasitology, serology and dipstick microhaematuria data by age group

Age group	Parasitology	Serology		Dipstick microhaematuria	
		Negative	Positive	Negative	Positive
1–5 years	Negative	25	17	9	33
	Positive	0	9	0	9
6–10 years	Negative	22	54	13	63
	Positive	2	61	4	59
	Total	49	141	26	164
